# Short-term effects of antiglaucomatous topical medication on the ocular surface in Wistar rats

**DOI:** 10.22336/rjo.2025.36

**Published:** 2025

**Authors:** Cristina-Mihaela Anghel-Timaru, Daniela Adriana Iliescu, Leon Zăgrean

**Affiliations:** 1 Division of Physiology-Neuroscience - Department of Functional Neurosciences “Carol Davila” University of Medicine and Pharmacy, Bucharest, Romania; 2 Ama Optimex Ophthalmology Clinic, Bucharest, Romania

**Keywords:** glaucoma, topical medication, ocular surface, Wistar rats, IOP = intraocular pressure, OSD = ocular surface disease, RE = right eye, LE = left eye, OS = ocular surface, IU = international unit

## Abstract

Topical treatment remains the first-line therapeutic approach for glaucoma, primarily aimed at reducing intraocular pressure (IOP), which is currently the only modifiable risk factor for the disease. However, while topical medications are effective in lowering IOP, they are also associated with a range of adverse effects.

This study evaluated the short-term side effects of various topical glaucoma medications in Wistar rats with experimentally induced intraocular hypertension. A glaucoma model was established using saline solution and viscoelastic pre-filled syringes, followed by the application of different topical anti-glaucomatous agents to assess both efficacy and potential side effects. Five groups of Wistar rats were utilized: four groups received other treatments, while the fifth group served as a control, with no therapy administered. The rats were monitored for 21 days following the induction of elevated intraocular pressure (IOP) and the commencement of treatment.

Preliminary results suggested that the number of drops administered per day might have a more significant impact on the outcomes than the presence or absence of preservatives in the eye drops.

## Introduction

The term “glaucoma” encompasses a group of ocular diseases characterized by progressive optic neuropathy, often leading to irreversible loss of visual acuity [1]. Due to the unclear etiology and pathogenesis of glaucoma, treatment strategies primarily focus on lowering intraocular pressure (IOP), the only modifiable risk factor currently available [2]. Topical medications remain the first-line treatment approach, aiming to reduce IOP to a level deemed sufficiently low to slow or halt the progression of the disease [3].

Several classes of medications are utilized in the form of topical antiglaucoma drops, including prostaglandins, beta-blockers, alpha-agonists, and carbonic anhydrase inhibitors, with additional agents under ongoing investigation showing promising results [4]. In many cases, a combination of two or more medications is necessary to achieve the desired therapeutic intraocular pressure (IOP). To improve patient adherence, fixed-dose combination therapies have been introduced, containing two antiglaucoma drugs in a single dispenser.

While topical treatments offer significant benefits, recent research has increasingly focused on the potential adverse effects of these medications on the ocular surface [5]. As glaucoma typically requires lifelong treatment, patient compliance is essential for maintaining quality of life and preventing vision loss. Various factors contribute to decreased adherence, with adverse drug effects being among the most significant [6]. Minimizing these effects may enhance patient compliance, thereby reducing the incidence of irreversible vision loss in affected individuals.

Ocular surface disease (OSD) is common among glaucoma patients receiving topical treatments, either coexisting with glaucoma or arising as a consequence of the therapy itself [7]. These effects may be attributed to the active ingredients in the medications or the preservatives used to prevent contamination and enhance drug penetration. In this context, preservative-free antiglaucomatous drops have gained attention, with studies suggesting that such formulations are less likely to induce adverse ocular surface effects [8-11].

This study aimed to assess and evaluate the impact of various anti-glaucomatous topical medications on the ocular surface in Wistar rats over a short-term treatment period.

## Materials and methods

### Materials

Among the materials used were: saline solution, 1 ml syringes, viscoelastic pre-filled syringes, topical ocular anesthetic, sevoflurane, chloral hydrate, cotton wool, I-care tonometer, blue light source, fluorescein, microscope, anti-glaucomatous topical medications (Preservative -free prostaglandin - latanoprost, Prostaglandin - latanoprost, Preservative-free combination -brinzolamide + brimonidine, fixed combination - dorzolamide + timolol).

### Glaucoma/Intraocular Hypertension model in Wistar rats

To induce an increase in intraocular pressure (IOP) in Wistar rats, two models were tested:

1. Intravitreal injection of saline solution;
2. Injection of a viscoelastic substance into the anterior chamber.

The intravitreal injection of saline solution was intended to create tension within the eyeball, resulting in slight compression of the episcleral vessels and a reduction in the elimination of aqueous humor, thereby increasing intraocular pressure (IOP). On the other hand, the injection of a viscoelastic substance into the anterior chamber aimed to partially block the trabecular meshwork, thereby impeding the drainage of aqueous humor and resulting in an increase in intraocular pressure (IOP).

To determine the most effective method for inducing elevated intraocular pressure (IOP), two nine-week-old Wistar rats, each weighing approximately 210 grams, were used. The following procedure was employed for a rat: 6 IU of saline solution (1 ml = 100 IU) was injected intravitreally into the right eye. At the same time, one drop of viscoelastic substance was introduced into the anterior chamber of the left eye. Before the procedure, the rat was anesthetized. Initially, sevoflurane, a volatile inhalant anesthetic, was applied by soaking cotton wool and placing it in a closed container, where the rat remained for 30 seconds to 1 minute to allow the anesthetic to take effect. Subsequently, an injectable anesthetic, chloral hydrate (1 gram of substance and 20 ml saline solution), was administered. The dosage was based on the rat’s weight: 0.8 ml of solution per 100 grams of body weight.

Following anesthesia, IOP measurements were obtained from both eyes using an I-care Pro tonometer, a portable device that employs disposable probes. The probe gently touched the eye to measure the intraocular pressure (IOP). Five measurements were taken, and the average value was recorded. If the difference between any two measurements exceeded two mmHg, additional measurements were taken until consistency was achieved. The initial IOP values obtained were as follows: right eye, 9.5 mmHg; left eye, 11.1 mmHg.

The injections were performed under a microscope, and before the procedure, a drop of Tropicamide 5% was instilled in both eyes to induce mydriasis.

For the right eye (RE), an intravitreal injection of saline solution was administered by inserting the needle posterior to the crystalline lens. For the left eye (LE), a drop of viscoelastic substance was introduced into the anterior chamber by inserting the needle through the cornea, approximately 2 mm from the sclero-corneal limbus.

Five minutes post-injection, IOP was measured again. For the right eye (RE), the IOP was 13.3 mmHg, while for the left eye (LE), it was 11.8 mmHg. The rats were then returned to their cages, and IOP was remeasured 5 days after injection. Five days later, the IOP for the right eye was 13.6 mmHg, and for the left eye, it was 11.5 mmHg.

To further confirm these results, a second Wistar rat was subjected to the same injection technique. For the right eye, 10 IU of saline solution was used: 8 IU were injected intravitreally, and 2 IU were injected into the scleral and episcleral regions when withdrawing the needle from the vitreous body. For the left eye, a viscoelastic substance was injected into the anterior chamber of the eye. The results were consistent with previous findings, with the right eye’s pressure measured at 13.5 mmHg and the left eye’s pressure at 9.2 mmHg, 7 days post-injection.

Thus, the intravitreal injection of 10 IU saline solution was established as the most effective method for creating a glaucoma model in this study.

To investigate the effects of topical therapy on the ocular surface, the Wistar rats were divided into five groups, each assigned a specific treatment regimen:

- Group 1: Prostaglandin treatment with preservative (latanoprost 50 μg/ml + BAK - benzalkonium chloride);
- Group 2: Prostaglandin treatment without preservative (latanoprost 50 μg/ml);
- Group 3: Fixed combination treatment without preservative (brinzolamide 1% + brimonidine 0.2% - 10 mg/ml + 2 mg/ml);
- Group 4: Fixed combination treatment with preservative (dorzolamide 20 mg/ml + timolol 5 mg/ml);
- Group 5: Control group (no treatment).

### Treatment procedure

All experiments were conducted in the Physiology and Neuroscience Laboratory. A total of 20 twelve-week-old Wistar rats, weighing between 180 and 200 grams, were randomly assigned to five groups, with four rats per group. All groups were maintained under identical environmental conditions and provided with the same diet.

To induce an increase in intraocular pressure (IOP), each rat was first anesthetized with sevoflurane, followed by an intraperitoneal injection of chloral hydrate, with the dosage adjusted according to the rat’s body weight (0.8 ml of solution per 100 grams of body weight).

**Fig. 1:**
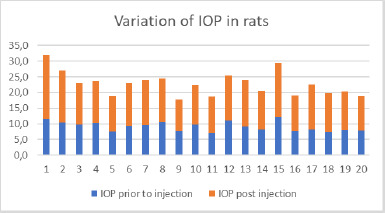
Variation of IOP in all 20 rats before and after injection

Upon anesthesia, IOP was measured in both the right and left eyes of each rat before injection. After administering the injection, the appearance of corneal haze was immediately noted, indicating an increase in IOP. Post-injection IOP measurements were then recorded. The pre- and post-injection IOP values are illustrated in **Fig. 1**.

The rats were left without treatment for 5 days to ensure that the effects of the injection remained stable.

Regarding the dosage of treatment, prostaglandin-containing topical medications were administered once daily, in the evening, while fixed-combination topical medications were administered twice daily, in the morning and evening. Treatment administration for groups 3 and 4 occurred in the morning between 6:00 and 8:00 AM, while for groups 1 through 4, administration was also in the evening between 6:00 and 8:00 PM. Group 5 served as the control group and received no treatment.

On days 7, 14, and 21, the rats were anesthetized with sevoflurane and an injectable anesthetic. IOP measurements were taken, and the anterior segment of the eye was examined under a microscope, with photographic documentation of the findings.

## Results

**Fig. 2:**
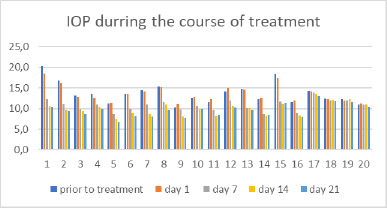
Variation of IOP during treatment

Regarding the reduction of intraocular pressure (IOP), the four groups that received treatment with topical hypotensive medications demonstrated a decrease in pressure, which approached baseline values. In contrast, the control group exhibited a slight, persistent increase in IOP at all measurement points (**Fig. 2**).

In Groups 1 and 2, IOP decreased by 27-40% from the initial values, showing effectiveness consistent with findings in published studies [1,2-14].

In Group 3, the reduction in IOP was somewhat less pronounced, with a decrease of 18-31% from the initial value, as reported in previous studies [15,16].

In Group 4, the brinzolamide/brimonidine fixed combination resulted in a 20-27% reduction in IOP, consistent with results from other studies [1,7].

Another parameter examined was the weight of the rats. Weight variation, specifically weight gain, was used as an objective measure to assess the quality of life and overall well-being of the animals. Before injection and treatment, the rats’ weights ranged from 180 to 200 grams, increasing to between 185 and 210 grams after 21 days. A more pronounced trend of weight gain was observed in Group 5. In contrast, the weight of rats in Groups 1-4 remained relatively stable, potentially due to the daily administration of treatment, which may have acted as a stressor.

Additionally, a noticeable behavior change was observed in the treated groups. These rats appeared more agitated and fearful compared to those in the control group, especially when the cage was moved. This behavioral response suggested that the rats might have anticipated the treatment and the discomfort it caused.

**Fig. 3:**
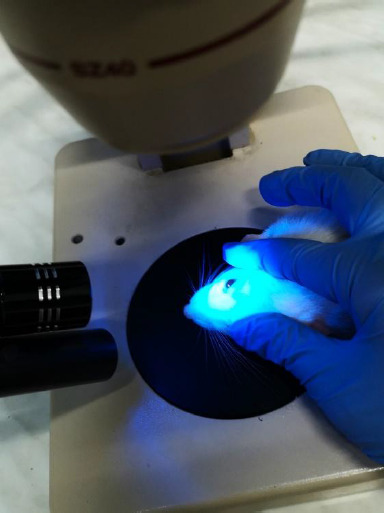
Rat placed under microscope for photographic evidence

For photo documentation, a microscope, a blue light source, fluorescein staining, and a Leica Camera (VARIO-SUMMILUX-H1:1.6-2.4/27-80 ASPH) were used to highlight possible epithelial defects and evaluate their effects on the ocular surface (**Fig. 3**).

To assess the effects of treatment on the ocular surface, evaluations were performed on the corneal epithelium, neovascularization, stromal edema, and any irregularities in the corneal surface that could be attributed to changes in tear film quality.

Each rat was stained with fluorescein and then examined under blue light. Any epithelial damage would cause fluorescein to adhere at the affected site, appearing as a green-yellow defect under blue light.

**Fig. 4:**
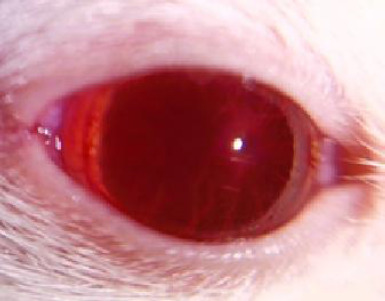
OS prior treatment

**Fig. 5:**
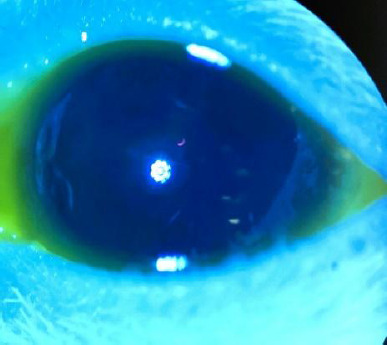
OS prior treatment -blue light

The condition of the cornea was documented both before and during treatment. Most rats showed no visible corneal defects before treatment, with no epithelial damage observed. The ocular surface (OS) appeared clear, smooth, and glossy, as evidenced in **Fig. 4, 5**.

There were two exceptions to this observation.

**Fig. 6:**
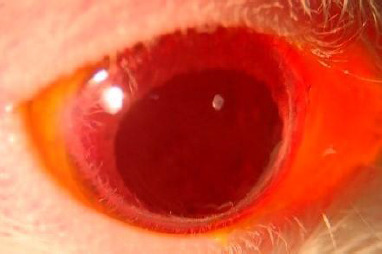
Peripheral neovascularization of the cornea in the rat

The first occurrence was in Group 3, before the initiation of treatment. As shown in **Fig. 6**, peripheral neovascularization of the cornea was observed in the upper sector. This was likely a result of the intravitreal injection of physiological saline and the subsequent induction of intraocular hypertension.

**Fig. 7:**
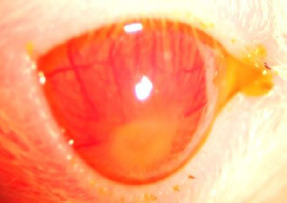
Rat on examination

The other notable exception occurred in Group 4. Five days after injection, before the initiation of treatment, one rat in Group 4 exhibited significant corneal edema, characterized by circumferential fluorescein staining, corneal neovascularization, and central corneal leucoma, as shown in **Fig. 7, 8**. The most likely cause of these findings was the induction of acute glaucoma following the injection, which likely resulted in angle closure.

**Fig. 8:**
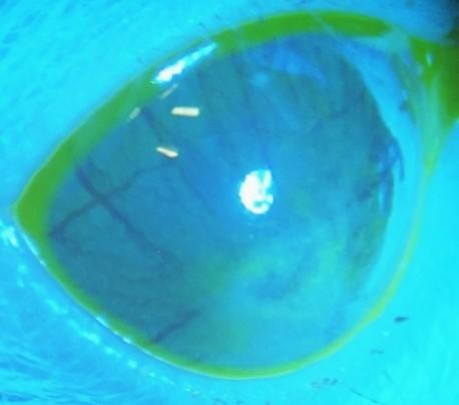
Rat exanimated under blue light and fluorescein staining

After treatment, changes were observed in the cornea, tear film, and conjunctiva of various rats.

**Fig. 9:**
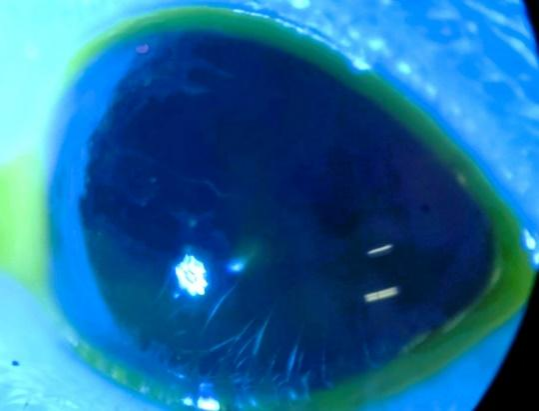
Rat from group 3, 21 days after starting treatment: no epithelial defects visible, but retention of fluorescein on ocular surface

In Group 1, most rats exhibited discrete retention of fluorescein, without localized epithelial defects. This was suggestive of a modified tear film and mild corneal epithelial dysfunction, as demonstrated in **Fig. 9**.

**Fig. 10:**
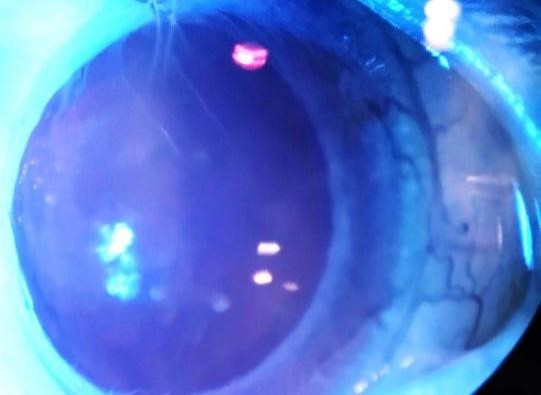
State of the rat from group 2 after 21 days of treatment

In Group 2, many rats exhibited fluorescein retention, with some showing pronounced conjunctival and episcleral vascularization, mild corneal haze, and even some corneal neovascularization, as shown in **Fig. 10**.

**Fig. 11:**
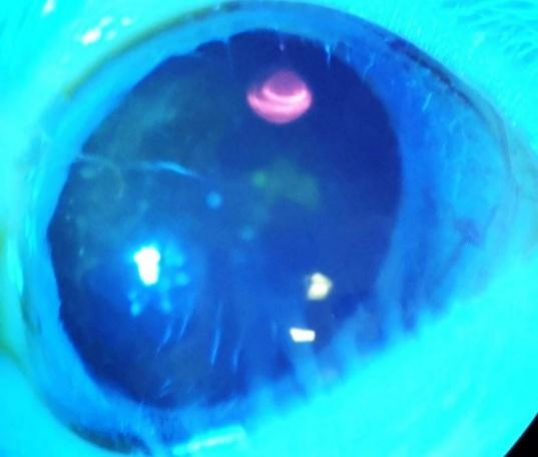
Central corneal staining and neovascularization in the rat in group 3 after 21 days of treatment

In Group 3, minor changes were observed in all rats, primarily diffuse fluorescein retention and mild corneal neovascularization. The most notable changes were observed in the rat that had exhibited peripheral corneal neovascularization before treatment. On day 21 of treatment, the neovascularization persisted, accompanied by conjunctival hyperemia. Additionally, diffuse central corneal staining was visible under blue light, as shown in **Fig. 11**.

**Fig. 12:**
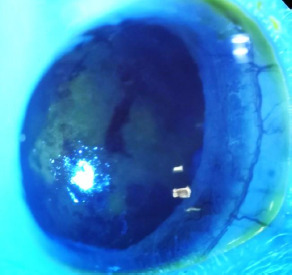
Rat from group 4 after 21 days of treatment - important central retention of fluorescein, with neovascularization

**Fig. 13:**
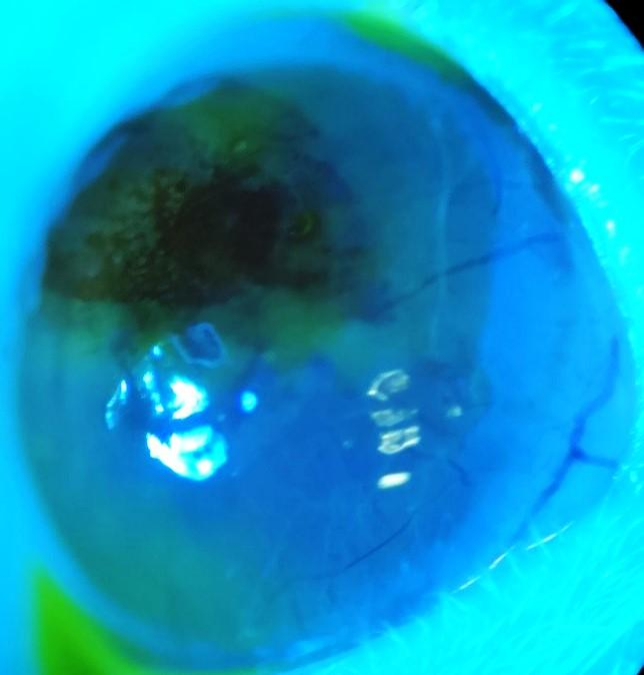
Increased neovascularization and evolution towards corneal ulcer in the rat in group 4

In Group 4, most rats exhibited corneal neovascularization, increased conjunctival neovascularization, and diffuse fluorescein retention, particularly in the central cornea, as observed in **Fig. 12**. The rat that had shown significant changes to the ocular surface before treatment experienced a worsening of the condition after treatment. The corneal leucoma progressed to a corneal ulcer, with extensive central fluorescein retention, and the neovascularization became visibly more pronounced, as shown in **Fig. 12, 13**.

In group 5, as no treatment was administered, no changes to the ocular surface were documented.

## Discussions

The impact of topical anti-glaucomatous therapy on the ocular surface is a topic of significant global interest. Numerous studies have contributed valuable insights into this issue, and ongoing efforts are focused on developing solutions to mitigate these effects and enhance the quality of life for patients who require such treatments.

In this context, the introduction of preservative-free anti-glaucoma medications to the market represents an important step toward addressing these concerns. Various studies suggest that these new formulations could significantly reduce adverse effects on the ocular surface and improve patient comfort, thereby enhancing overall quality of life.

The present study aimed to evaluate the short-term effects of anti-glaucomatous medications on the eyes of Wistar rats. The primary objective was to determine whether the active substance in the medication or the preservative used contributed more significantly to these adverse effects.

Upon evaluating the four groups treated with anti-glaucoma medications, no significant differences were observed in terms of ocular surface effects. Both groups receiving preservative-containing treatments and those with preservative-free formulations exhibited various ocular surface changes, including corneal haze, diffuse or localized epithelial defects, neovascularization, and conjunctival hyperemia. However, no clear differences were observed that could either support or refute the prevailing hypothesis attributing a primary role to the preservative in inducing adverse effects.

Weight gain was a parameter observed in this study. While slight differences were observed between the groups, the most significant finding was the more pronounced weight gain in Group 5, the control group, in comparison to the other four groups. This suggested that the stress induced by the treatment might have a substantial impact on the animals’ quality of life.

## Conclusion

It is important to note that the results of this study cannot be generalized due to the small sample size and the inability to assess the ocular comfort levels of the rats during the treatment period. Additionally, other topical medications that could potentially mitigate these adverse effects, such as artificial tears containing hyaluronic acid, were not included in this study.

The next logical step is to conduct a study focusing on the quality of life and the adverse effects of various topical anti-glaucomatous treatments in human patients. We hypothesized that it was not solely the preservative that produced these effects, but rather the cumulative impact of the preservative combined with the active medication. Specifically, we propose that more frequent daily applications could lead to more pronounced adverse effects.
